# Chronic recurrent multifocal osteomyelitis in association with pyoderma gangraenosum

**DOI:** 10.1186/s12903-016-0275-z

**Published:** 2016-09-01

**Authors:** Matthias Christian Wurm, Ines Brecht, Michael Lell, Kathrin Brunner, Konstantinos Theodorou Mitsimponas, Martin Chada, Julia Jahn, Friedrich-Wilhelm Neukam, Cornelius von Wilmowsky

**Affiliations:** 1 Department of Oral and Maxillofacial Surgery, University of Erlangen-Nürnberg, Glückstrasse 11, 91054 Erlangen, Germany; 2 Department of Paediatrics and Adolescent Medicine, University of Erlangen-Nürnberg, Erlangen, Germany; 3 Department of Radiology, University of Erlangen-Nürnberg, Erlangen, Germany; 4 Department of Pathology, University of Erlangen-Nürnberg, Erlangen, Germany; 5 Department of Maxillofacial Surgery, Royal Free Hospital, London, UK; 6 Department of Dermatology, University of Erlangen-Nürnberg, Erlangen, Germany

**Keywords:** Chronic recurrent multifocal osteomyelitis, CRMO, Non-bacterial osteomyelitis, NBO, Pyoderma gangraenosum

## Abstract

**Background:**

Chronic recurrent multifocal osteomyelitis (CRMO) is a rare acquired inflammatory skeletal disorder of unknown origin. CRMO was first described by Gideon in 1972 and mainly affects children and young adults of female gender. The CRMO is part of the clinical picture of non-bacterial Osteomyelitis (NBO) and typically presents a relapsing recurring course with both remission and spontaneous exacerbation. CRMO is typically encountered in the limbs and the metaphysis of long bones in particular. Usually the clinical symptoms include painful swellings of the affected regions. This case report describes the rare case of a CRMO of the mandible in association with pyoderma gangraenosum.

**Case presentation:**

A 14-year old female caucasian patient, residing in the south of Germany, presented in the oncological outpatient clinic of our Department of Paediatrics and Adolescent Medicine in June 2014 complaining of increasing neck pain and progressive swelling at her left cheek ongoing for about 6 weeks. These symptoms had been occurring quarterly for 4 years, but had never been as pronounced. Blood biochemistry showed a moderately elevated CRP (35 mg/l) and a significantly increased blood sedimentation rate (BSR 48/120 mm). The panoramic radiograph, however, revealed a bone alteration in the left mandibular region. Further investigations confirmed the diagnosis of CRMO.

**Conclusion:**

The present case underlines the fact that rare diseases might occasionally present with even more rare symptoms. These occasions can obviously be considered to present a considerable diagnostic challenge.

## Background

Chronic recurrent multifocal osteomyelitis (CRMO) is a rare acquired inflammatory skeletal disorder of unknown origin. CRMO was first described by Gideon in 1972 [1] and mainly affects children and young adults of female gender [2–4]. Very similar to CRMO is the SAPHO-Syndrome, whose hallmarks are the presence of synovitis, acne, pustulosis, hyperostosis and osteitis (SAPHO). SAPHO-Syndrome is considered to affect predominantly the adult population. The age of the patient is, however, not diaforodiagnostic [5], since adults with CRMO and children with SAPHO-Syndrome have been described so far [6, 7].

The CRMO is part of the clinical picture of non-bacterial Osteomyelitis (NBO) and typically presents a relapsing recurring course with both remission and spontaneous exacerbation. CRMO is typically encountered in the limbs and the metaphysis of long bones in particular. Usually the clinical symptoms include painful swellings of the affected regions. This case report describes the rare case of a CRMO of the mandible in association with pyoderma gangraenosum.

## Case presentation

**Fig. 1:**
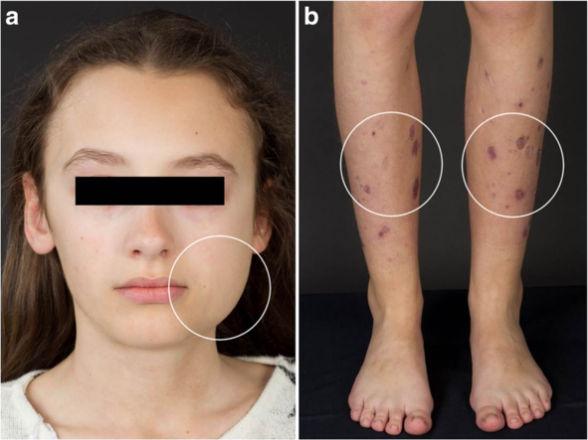
**a** Distinct swelling of the left cheek (*circle*). **b** Skin lesions on both lower limbs (*circles*)

A written consent for this case report has been obtained by the parents. A 14-year old female caucasian patient, presented in the oncological outpatient clinic of our Department of Paediatrics and Adolescent Medicine in June 2014 complaining of increasing neck pain and progressive swelling at her left cheek about 6 weeks ago (Fig. 1a). These symptoms had been occurring quarterly for 4 years, but had never been as pronounced. In all previous occasions the symptoms had improved with the use of ibuprofen. Furthermore skin lesions were noticed on both lower legs. The patient told us that these lesions had persisted for 3 years and had usually improved during the summer (Fig. 1b).

Suffering from ongoing trismus, the patient consulted an ear nose throat (ENT) specialist 4 months ago. The initial work diagnosis was that of a parotitis and an antibiotic therapy was implemented. Due to the persistence of the symptoms the advice of a cranio-maxillo-facial surgeon (CMFS) was sought, so as to investigate a possible relation of the symptoms to the wisdom teeth. Pericoronitis or any other disease related to wisdom teeth could indeed be excluded with the help of clinical examination and radiological investigation (PR - panoramic radiograph).

**Fig. 2:**
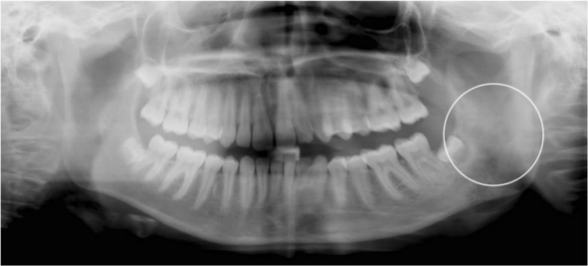
Alteration of the trabecular structure of the left mandibular angle (*circle*)

**Fig. 3:**
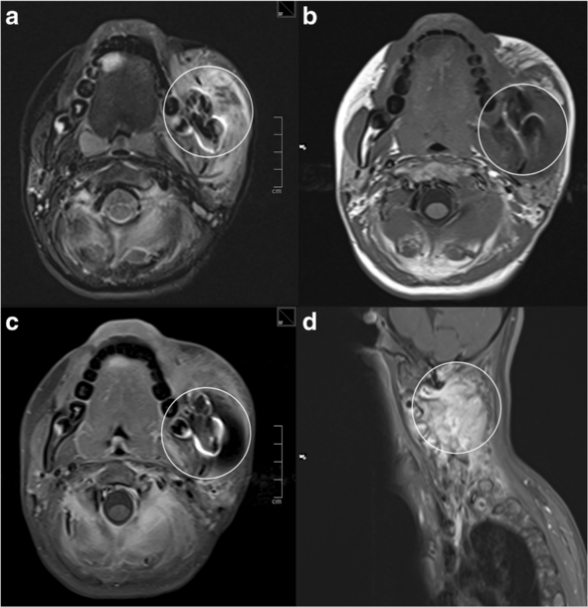
MRI-T2: Soft-tissue tumour with infiltration of the surrounding bone located at the left corpus and ramus of the lower jar (**a**, **b**, **c** - *circles*). Similar osseous and soft tissue lesions were detected in the cervical vertebrae 2–5 (**d**) (*circle*) including the vertebral arches and the transverse processes

The PR, however, revealed an alteration of the trabecular structure of the left mandibular angle (Fig. 2). A Magnetic Resonance Imaging (MR) was performed for further investigation. Suspicion for Langerhans Cell histiocytosis (LCH), Ewing sarcoma, fibrous dysplasia, lymphoma and osteomyelitis was risen (Fig. 3). The young patient was referred to our department of paediatric oncology.

An initial assessment of the patient revealed a good general but a deteriorated nutritional status. The skin of the lower legs showed multiple ulcerous blueish skin lesions (Fig. 1b) and the skin of the face and the upper part of the body presented several acne scars. A 7x5cm, hardened, tender on palpation mass was detected at her left cheek. No clear local signs of inflammation were present. The lymph node status was unremarkable.

Further clinical examination revealed an abnormal posture, characterized by inclination of the head to the left, elevated shoulders and scoliosis. Pain was elicited on percussion of the cervical spine. Blood biochemistry showed a moderately elevated CRP (35 mg/l) and a significantly increased blood sedimentation rate (BSR 48/120 mm).

**Fig. 4:**
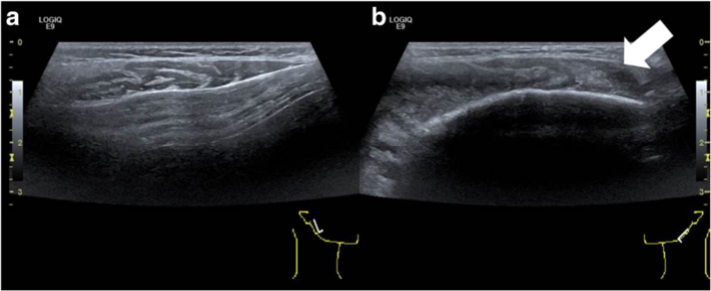
Ultrasound of the soft tissue of the cheeks (**a**, **b**). Inhomogenous widening on the left side (**b**) (*circle*)

Ultrasound of the head and neck showed submandibular cervical lymphadenopathy at the left side and a diffuse swelling affecting the soft tissues of the left cheek (Fig. 4).

On chest x-ray (pa), no suspicious pulmonary nodules or consolidations were found.

**Fig. 5:**
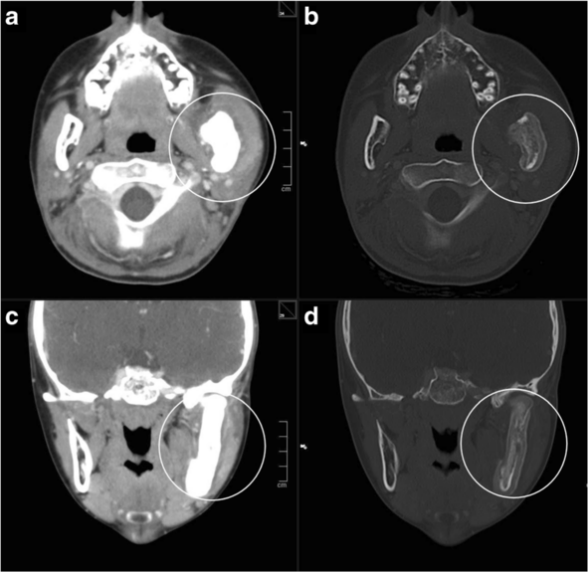
**a**, **c** The CT reveals an extensive thickening of the left mandible with contrast agent enhancing soft tissue mass (*circles*). **b**, **d** Destruction of the cortex of the mandible and massive periostal reaction. Similar changes can also be found in the posterior aspect of the cervical spine (*circles*)

Further imaging with contrast-enhanced CT was performed which revealed an extensive thickened left corpus and ramus of the mandible with a soft tissue mass (Fig. 5a). Destruction of the cortex of the mandible and a massive periosteal reaction (Fig. 5b), a left-sided cervical lymphadenopathy and diffuse contrast-enhancement in the soft tissue surrounding the mandible were identified. Similar osseous and soft tissue lesions were detected in the cervical vertebrae 2–5 including the vertebral arches and the transverse processes.

**Fig. 6:**
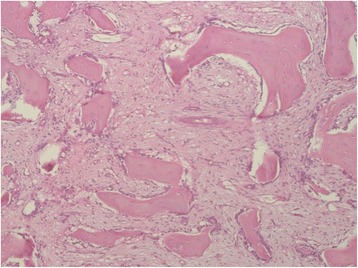
Biopsy from the lower jaw: Features of a benign fibro-osseous lesion, consistent with fibrous dysplasia or secondary changes after osteomyelitis. HE 100×

Biopsies were taken from both the lower jaw and the skin lesions. The histological examination of the specimen from the lower jaw showed features of a benign fibro-osseous lesion, consistent with fibrous dysplasia or secondary changes after osteomyelitis (Fig. 6). GNAS mutational analysis was performed to definetly exclude FD. The dermatopathological examination showed an abscess-forming neutrophil inflammation; taking into consideration the clinical appearance, the findings were consistent with pyoderma gangraenosum (PG).

**Fig. 7:**
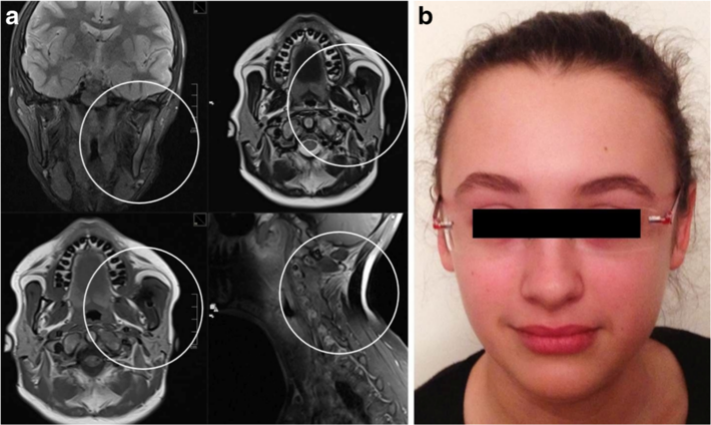
MRI (**a**) and the patient (**b**) 6 months after therapy. The MRI shows a distinct decrease of the thickening of the left mandible and the soft tissue mass in the posterior aspect of the cervical spine (*circles*)

Treatment with Ibuprofen and Sulbactam/Amoxicillin led to significant decrease of the mandibular swelling. Furthermore the pain in the mandible and the back subsided, resulting in regular posture. An obvious reduction of the lower limb skin lesions and of acne vulgaris as well as increasing appetite were observed (Fig. 7).

After taking into consideration the clinical findings, the results of the performed investigation and the response to the provided treatment, the diagnosis of a chronic recurrent multifocal osteomyelitis (CRMO) was made.

The CRMO is part of the clinical picture of non-bacterial Osteomyelitis (NBO) which can be classified into three progression forms - acute NBO, persistent chronic NBO or chronic recurrent multifocal osteomyelitis [8]. CRMO typically presents a relapsing recurring course with both remission and spontaneous exacerbation [9, 10]. Intermission could last from months to years [11].

Jansson proposed major and minor diagnostic criteria of NBO (Table 1) [8]. NBO is quite likely if two major or one major and three minor criteria are met. Jannson also showed that the fewer criteria are met the more likely is an acute NBO. Furthermore the more criteria are met the more likely is a CRMO.

**Table 1:** Major and minor diagnostic criteria of NBO

Major diagnostic criteria	Minor diagnostic criteria
Radiologically proven osteolytic/-sclerotic bone lesion	Normal blood count and good general state of health
Multifocal bone lesions	CRP and ESR mildly-to-moderately elevated
PPP or psoriasis	Observation time longer than 6 months
Sterile bone biopsy with signs of inflammation and/or fibrosis, sclerosis	Hyperostosis
	Associated with other autoimmune diseases apart from PPP or psoriasis
	Grade I or II relatives with autoimmune or autoinflammatory disease, or with NBO

CRMO is a diagnosis that is made by exclusion. Medical history, radiological diagnostics and a bone biopsy are the cornerstones for establishing the diagnosis of CRMO. According to the current AWMF-guideline [12] numerous diagnostic methods might be used to exclude malignant or inflammatory conditions. The exclusion of malignant processes has the highest priority.

CRMO is typically encountered in the limbs and the metaphysis of long bones in particular. Usually the clinical symptoms include painful swellings of the affected regions. None of the typically affected sites were involved in our patient; two rare sites – the lower jaw and vertebral bodies – were affected instead. Radiological signs are hyperostosis and osteitis with lightening of the affected areas and surrounding sclerosis. As in children multifocal bone lesions could be consistent with a good number of differential diagnoses–e.g. Langerhans cell histiocytosis or leukemia [13], a biopsy is recommended [14]. Ewing sarcoma could not radiologically be excluded since it might look identically. An exclusion of this diagnosis could only be achieved through the following biopsy.

Histologically CRMO shows characteristics of inflammation and/or fibrosis without a detectable pathogen [13, 14]. In our patient the pattern of a benign fibro-osseous lesion was present consistent with an expired inflammatory process. Fibrous dysplasia could be excluded from the differential diagnosis since a negative GNAS1-mutational status (GNAS 1 wild type) was identified. The histomorphological pattern was consistent with changes after osteomyelitis or previous surgical procedure. As there were no earlier interventions CRMO became more likely.

Jansson determined that in 31 % of CRMO patients exoskeletal findings were present [8]. An association with autoimmune disorders as IBD (chronic inflammatory bowel disease) [2] respectively Crohn’s disease [15] or Takayasu arteriitis [16] is described. Far more frequent - incidence varies from 23 to 80 % [7]- associated skin lesions are present – as in our patient. Not only psoriasis and palmoplantar pustolosis [13] but also pyoderma gangrenosum [2] have been described so far. However various autoimmune diseases are considered as associated skin lesions but as they also occur alone, it can easily happen that these lesions are not included in the diagnosis -making [17–20]. Our patient also presented dubious skin lesions consistent with pyoderma gangraenosum. Dagan et al. wrote a case report of a CRMO in the ninth and tenth ribs as well as the tibia with associated PG. Nurre et al. presented a case of CRMO with associated PG. However CRMO presented initially at the left hand as well as other bones except the mandible. After biopsy a PG presented at the site of biopsy. Innis described a similar case with involvement of several bones. In his case, PG was present at the scalp and the limbs. In an article of Edwards et al. CRMO manifested at both tibiae and associated PG. To our knowledge this is the first of such case –involvement of the mandible with associated PG- described in the literature.

Due to the fact that its pathogenesis remains unclear, its treatment is mainly symptomatic. Anti-inflammatory drugs form the basis of the current treatment concept; occasionally the use of cortisone therapy might be necessitated in severe cases [4, 15, 21]. Our patient showed a good response to anti-inflammatory drugs.

Bisphosphonates like pamidronate showed initial promising results since they act both as anti-resorptive and anti-inflammatory agents [22]. TNFα-inhibitors seem to be effective as well but as Costa-Reis stated there is no consensus about this regime so far [23, 24]. In severe cases limited surgical interventions or orthopedic procedures might be helpful or even necessary. Physiotherapeutic treatment might reduce the symptoms when the spine is affected.

The prognosis is unclear so far. Results from studies vary from complete remission to symptomatic courses for as long as 16 years [3, 13].

By reflecting on the NBO criteria and how they applied in our case it becomes apparent that this rare disease has in this instance manifested in an even more unusual way. Involvement of the lower jaw is very rare and accounts for about 5 % of the cases [7]. Involvement of the spine accounts about 30 % of the cases. Pathological fractures are described in the literature [8]. Concurrent appearance of CRMO and pyoderma gangrenosum is mentioned sporadically in the literature so far [2, 16, 25]. The combination of multifocal bone lesions at atypical sites and PG has obviously encumbered our diagnostic efforts. A biopsy to confirm or exclude LCH, Ewing sarcoma, fibrous dysplasia and lymphoma was mandatory. Exclusion of fibrous dysplasia was supported by GNAS1 mutational analysis which revealed the presence of the wild type sequence, rendering this diagnosis unlikely. The signs of fibrosis that were detected were also consistent with CRMO. Therefore three of four major criteria were met even though the sites of the skeleton that were affected were atypical.

The patient’s work-up showed that three minor criteria were also met: Blood biochemistry revealed a normal blood count; CRP and ESR were moderately elevated whilst the patient presented a good general state of health. Observation time was longer than 6 months.

Corresponding to recent studies the patient showed a good response to Ibuprofen.

## Conclusion

The present case underlines the fact that rare diseases might occasionally present with even more rare symptoms. CRMO in the mandible and CRMO with associated PG have already been described. For the first time we described an involvement of the mandible with associated PG. These occasions and combinations can obviously be considered to present a considerable diagnostic challenge. An in depth knowledge about the disease and its symptoms is a matter of extreme value in such cases.

In addition, an accurate medical history is of paramount importance when uncertain changes of the cranial skeleton are present. The physician should always enquire about new osseous changes, pain or new lesions and additional questions e.g. SCHOLAR [26]. If uncertain skin lesions or rheumatic disorders are present, CRMO should be in mind. NBO criteria might be helpful for diagnosis. As there are various manifestations, an interdisciplinary approach can be useful.

## Abbreviations

BSR/ESR: Blood sedimentation rate
CMFS: Cranio-maxillo-facial surgeon
CRMO: Chronic recurrent multifocal osteomyelitis
CRP: C-reactive protein
CT: Computed tomography
ENT: Ear nose throat specialist
FD: Fibrous dysplasia
IBD: Chronic inflammatory bowel disease
LCH: Langerhans cell histiocytosis
MR(I): Magnetic resonance imaging
NBO: Non-bacterial osteomyelitis
PG: Pyoderma gangraenosum
PR: Panoramic radiograph
SAPHO: Synovitis, acne, pustulosis, hyperostosis and osteitis
